# Efficient Hydrothermal Synthesis of SSZ-13 with Variable Grain Size

**DOI:** 10.3390/ma13081829

**Published:** 2020-04-13

**Authors:** Yibao Wang, Chen Wang, Jun Wang, Jianqiang Wang, Lei Wang, Cheng Xu, Meiqing Shen

**Affiliations:** 1 State Key laboratory of Engine reliability, Weifang 261061, China; wangyib@weichai.com (Y.W.); wanglei@weichai.com (L.W.); xuc@weichai.com (C.X.); 2 Weichai Power Co., Ltd., Weifang 261061, China; 3 Key Laboratory for Green Chemical Technology of State Education Ministry, School of Chemical Engineering & Technology, Tianjin University, Tianjin 300072, China; chenwang87@nuc.edu.cn (C.W.); wangjun@tju.edu.cn (J.W.); jianqiangwang@tju.edu.cn (J.W.); 4 School of Environment and safety Engineering, North University of China, Taiyuan 030051, China

**Keywords:** SSZ-13, hydrothermal synthesis, different grain sizes, seeding method

## Abstract

To meet the industrial needs for SSZ-13, variable sizes of SSZ-13 with different Si/Al ratios were firstly obtained by conventional hydrothermal synthesis using the seed method. Using a set of characterizations, like X-ray fluorescence (XRF), X-ray diffraction (XRD), and scanning electron microscopy (SEM), the physicochemical structure and size distribution could be traced. After the specific Si/Al ratio of SSZ-13 zeolites was optimized, synthesized by changing the amounts of structure-directing agents (SDAs) and NaOH, the obtained SSZ-13 showed a high degree of crystallinity. With the limitation of the pH values, the variation of the alkalinity and water content was not helpful to generate different grain sizes of SSZ-13 materials. With the help of ground seed, the various grain sizes of SSZ-13s from 0.4 to 4 μm had a similar degree of crystallinity and size distribution. Moreover, due to the identical intensity of the Al peak in the NMR results, the different grain sizes of SSZ-13s had the same acidity. Our study revealed that using the seed method was an easy and efficient way to synthesize SSZ-13s with different sizes.

## 1. Introduction 

Academia and industry have been highly concerned in the last decade with silicoaluminate zeolites of SSZ-13 with the chabzite (CHA) structure, showing the same topological structure as SAPO-34. The SSZ-13 material contains 4-, 6-, and 8-membered ring (MR) units to form a three-dimensional pore structure with a large 7.3 Å cage and 3.8 Å 8-MR windows [1]. Due to the unique pore system, SSZ-13 has excellent low-/high-temperature hydrothermal stability, and the production selectivity can be widely used in the methanol-to-olefin (MTO) process and NOx removal technology (NH_3_-selective catalytic reduction (SCR)) in the diesel industry [2,3,4,5]. 

According to the informed studies on CHA zeolites, the grain size may influence mass transfer restrictions, and this will in turn influence the catalytic behavior. Some products generated in the methanol-to-olefin (MTO) reaction occupy the entire cage and result in poor catalytic activity [3], while this phenomenon does not occur in the NH_3_-SCR reaction, as all reactants have a smaller diameter than that of the pore size of CHA [6]. Besides that, the grain size could lead to different hydrothermal stabilities and lifetimes. Huang et al. [7] found that the small size of SAPO-34 with a low density of surface acidity and the CHA structure was relatively stable after low-temperature hydrothermal aging. In the reaction of MTO, the small size of SSZ-13 or SAPO-34 results in a long lifetime as the small size suppresses phenanthrenes’ generation and decreases the coke formation rate [5,8]. In addition, it is reasonable to believe that the small size of zeolites on a honeycomb carrier may increase the backpressure, and that would hinder the reaction process. Taking all these facts into account, the controllable synthesis of variable sizes of SSZ-13 according to the actual situation is an efficient way to cope with all the possible operating conditions in the real situation.

Actually, many methods have been developed to prepare different sizes of CHA zeolites. Regarding SAPO-34 materials, Song et al. [9] found that the size of SAPO-34 could be varied from 0.1 to 1.5 μm under microwave radiation. Wang et al. [10] used the ammonium-type organosilane surfactant Trimethoxysilyl)propyl]octade-cyldimethylammonium chloride (TPOAC) and the template diethylamine to synthesize elliptical-like SAPO-34 aggregates, consisting of 50 nm particles. Wang et al. [11] used a two-step hydrothermal process, with a relatively low-temperature hydrothermal crystallization followed by a high-temperature hydrothermal crystallization, to produce 0.16–0.55 μm SAPO-34 crystallites. Yang et al. [12] used a top-down approach, combining post-synthesis milling and recrystallization, to obtain nanoscale Cu/SAPO-34 particles of 0.05 to 0.35 μm. For SSZ-13 zeolites, adding organic compounds into the synthetic precursor solution of SSZ-13 is a common method to generate small-sized SSZ-13 particles. Li et al. [13] added the large molecular surfactant hexadecyl trimethyl ammonium bromide (CTAB) into conventional gel, called a one-pot synthesis procedure, to obtain nano-sized SSZ-13 zeolites. Xu et al. [5] used anionic and cationic organic compounds, polyacrylamide (APAM) and polyacrylamide (CPAM), to produce nano-SSZ-13 zeolites successfully. Hereto, even though some contributions have achieved the generation of small-sized CHA zeolites, the following aspects also deserve much attention. The mentioned synthesis methods usually require another process, and some processes or additives are quite complex and have a high cost, which are not suitable for commercial production. Additionally, the size of most synthesized particles is small, and the size distribution is not uniform, indicating that the obtained particles may easily aggregate and may not be appropriate for real applications. In short, it is important to perform further studies on an easy and efficient method to synthesize different sizes of CHA with a uniform size distribution.

Here, different sizes of SSZ-13 with a uniform size distribution were investigated. Recently, relying on the successful experience in the synthesis of different sizes of Cu/SAPO-34 with a uniform size distribution [7,14], we for the first time obtained different sizes of cubic-like SSZ-13 particles with a uniform size distribution (Si/Al = 25) [6]. In the present work, we will provide more data and experimental details, using X-ray fluorescence (XRF), X-ray diffraction (XRD), and scanning electron microscopy (SEM), to corroborate further that this method can be used to synthesize the different sizes of SSZ-13 with a uniform size distribution with different Si/Al ratios (8, 15, and 25). Notably, in this work, Na/SSZ-13s were firstly synthesized and H/SSZ-13s prepared via ammonia exchange and calcination to probe the above viewpoints.

## 2. Experimentation

### 2.1. Catalyst Preparation

Na/SSZ-13 synthesis: The material sources for Si and Al were silica sol (25 wt% SiO_2_, Qingdao Jiyida Silica Reagent Factory, Qingdao, China) and pseudoboehmite (68 wt% Al_2_O_3_, Shandong Aluminium Industry Co., Ltd., Zibo, China), respectively. TMAda-OH (25 wt%, Sinopec Qilu Petrochemical Co., Ltd., Zibo, China) was used as the templating agent. The gel was firstly mixed with SDA, NaOH liquid, and pseudoboehmite while stirring for 1 h. Then, silica sol was added into the mixture while stirring for another 1 h. Na/SSZ-13 was prepared using the following composition: 1 SiO_2_: a (0.06/0.03/0.02) Al_2_O_3_: b (0.025/0.05/0.1/0.15) Na_2_O: c (0.05/0.1/0.15/0.2) SDA: d (20/30) H_2_O. The resulting gel was then sealed into a 200 mL Teflon-lined stainless steel autoclave and placed in an oven at 160 °C for four days. After the crystallization process, the solid was separated from the mother liquid via centrifugation and washed with deionized water. Finally, the powder was dried in an oven at 100 °C for 12 h and calcined in a muffle furnace with air at 650 °C for 8 h to remove the template agent. Before characterization, H/SSZ-13 was prepared via ammonia exchange and calcination. The nomenclatures of the samples in Section 2.1 and Section 2.2 are S-x, H-y, respectively, where x stands for the number of experiments (1 to 17) and y stands for the grain size of SSZ-13 under a fixed Si/Al ratio according to the SEM results.

Cu/SSZ-13s were selected to synthesize to confirm the exchange capacity, and the synthesis process is listed in the following: H/SSZ-13 was further exchanged in a 1 M (NH_4_)_2_SO_4_ solution at 80 °C for 4 h and 0.5 M Cu(NO_3_)_2_ at 80 °C for 1 h, respectively. The Cu/SSZ-13s were finally obtained by calcining at 550 °C for 6 h in a muffle furnace.

### 2.2. Catalyst Characterization

The Si and Al concentrations of the as-prepared SSZ-13 materials were measured by X-ray fluorescence (XRF) spectroscopy. The cooper content of the obtained Cu/SSZ-13 catalysts was analyzed by inductively coupled plasma and atomic emission spectrometry (ICPAES). 

Two models of Scanning electron microscopy (Hitachi Limited/FEI, Tokyo/Portland, Japan/USA) images were taken using a field emission microscope. Prior to scanning, a sample was pasted on carbon tape and then covered with an Au film for better electrical conductivity. 

The XRD spectra were collected using an X’ Pert Pro diffractometer (Bruker Daltonics, Karlsruhe, Baden-Württemberg, Germany) with nickel-filtered Cu Kα radiation (λ = 1.5418 A) and operated at 40 kV and 40 mA in the range of 5–50° with a 0.01° step-size. The relative crystallinity was independently normalized at each Si/Al ratio using the total areas of five peaks (201, 003, 211, 104, and 220 phase) compared with the highest crystallinity sample.

^27^Al NMR measurements were conducted on a Varian Infinity plus 300WB Spectrometer (VARIAN, Palo Alto, State of California, U.S), and a Al(NO_3_)_3_ aqueous solution was used as a reference for ^27^Al NMR spectroscopy. Notably, the samples were pretreated at 300 °C under a vacuum for 12 h to remove water prior to measurements.

## 3. Results and Discussion

In this work, two methods, formula optimization and controllable preparation, are involved to illustrate how the variable sizes of SSZ-13s with a uniform size distribution in wide range of Si/Al ratios are obtained. 

### 3.1. Formula Optimization for the Synthesis of Different Si/Al/SSZ-13s 

It is well known that Na/SSZ-13 materials with a wide range of Si/Al ratios are usually obtained via hydrothermal methods using Al(OH)_3_ and colloidal silica with TMAda-OH (N,N,N-trimethyl-1-adamantammonium hydroxide) as the structure-directing agent (SDA) present in the NaOH solution [1]. Due to the importance of SDAs and NaOH for SSZ-13 materials [6,15], the SDAs related to the NaOH content in the precursor were considered in this study. 

The optimal synthesis condition can be obtained using the designed experimental matrix that contained variable parameters of the compositions of SDAs and NaOH. Three Si/Al ratios (8, 15, 25) were selected as the representative and general experimental variables, as seen in Table 1. From the viewpoint of the framework for the generation of aluminosilicates, some Al^3+^ substitute a fraction of the lattice Si^4+^, depending on the content of the SDAs. Besides that, the NaOH solution provides the extra lattice cations and extra -OH, which compensate the anionic charges of the SSZ-13 materials obtained to synthesize the gel. Thus, the crystallization of SSZ-13 should generate a particular range of molar ratios in the synthesized gel. 

**Figure 1:**
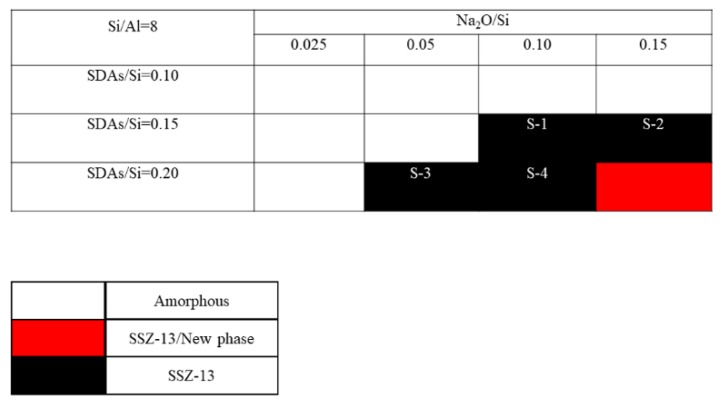
The crystallization of SSZ-13 with Si/Al = 8 as a function of the experimental variables in alkaline media. SDAs, structure-directing agents.

**Figure 2:**
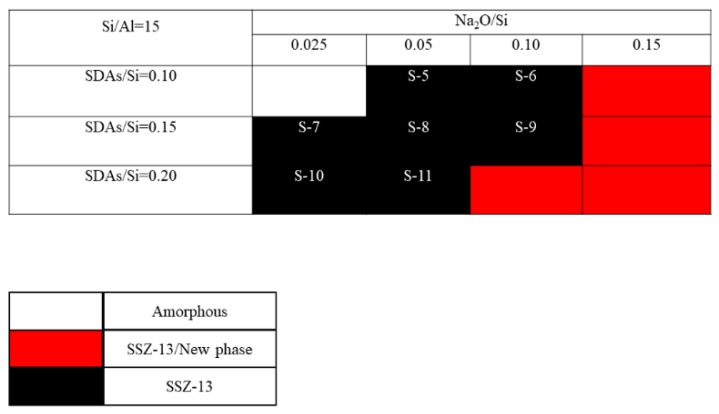
The crystallization of SSZ-13 with Si/Al = 15 as a function of the experimental variables in alkaline media.

**Figure 3:**
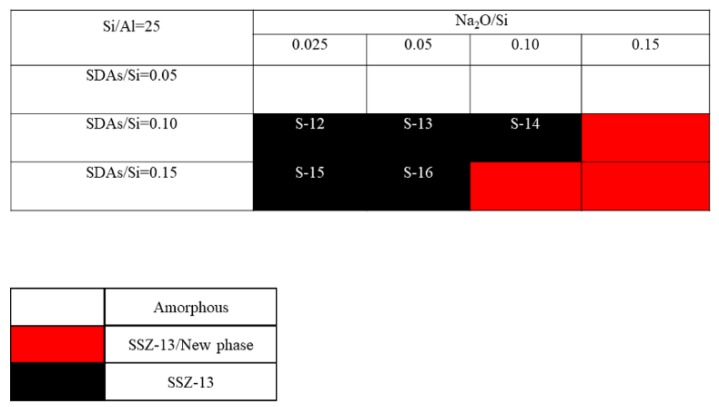
The crystallization of SSZ-13 with Si/Al = 25 as a function of the experimental variables in alkaline media.

The crystallization of SSZ-13 with Si/Al = 8 under different molar ratios of NaOH and SDAs was firstly probed, and the results are shown in Figure 1. Less SDA and NaOH in the gel were unfavorable for the crystallization of SSZ-13 materials; moreover, the SSZ-13/new phase was generated when the content of NaOH in the gel was too high. Conversely, when SDA/Si was from 0.15 to 0.2 and Na_2_O/Si was from 0.05 to 0.15, only the SSZ-13 materials with Si/Al = 8 could be obtained (black squares). Actually, the pH of all the successfully synthesized gels was measured before the gel was sealed into the Teflon-lined stainless autoclave, and the suitable pH levels were from 13.0 to 13.8. Accordingly, sufficient SDAs and suitable pH values were essential factors to determine the synthesis of SSZ-13, which strongly supported the above conclusion about the synthesis of aluminosilicates zeolites. With the increase of the Si/Al ratio of the synthesized SSZ-13, the required molar ratios of SDAs/Si and Na_2_O/Si decreased because the content of Al ions in the gel decreased, as shown in Figure 2 and Figure 3. Again, the crystallization of SSZ-13 required suitable conditions. However, it must also be mentioned that the molar ratio of SDAs/Si was equal to 0.05, which was too low to synthesize SSZ-13 with Si/Al = 25 in our work.

**Figure 4:**
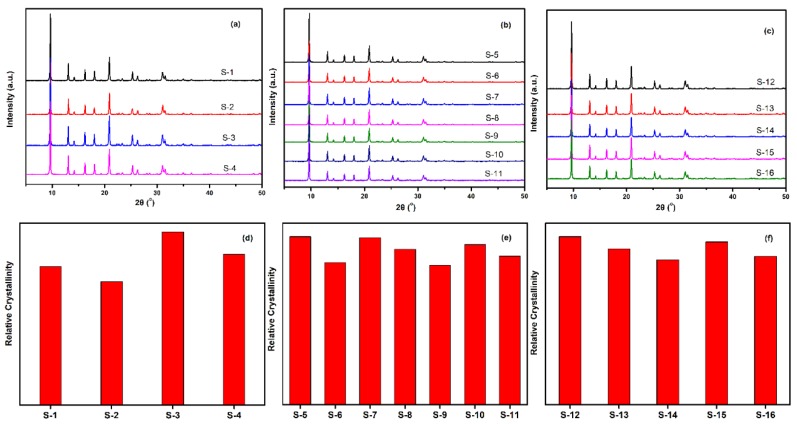
XRD pattern (top) and relative crystallinity (bottom) of SSZ-13 with different Si/Al ratios. (**a**,**d**) for Si/Al = 8 SSZ-13 materials; (**b**,**e**) for Si/Al = 15 SSZ-13 materials; (**c**,**f**) for Si/Al = 25 SSZ-13 materials. S-1, Sample 1.

The crystalline SSZ-13 materials of different Si/Al ratios were measured by XRD, and the results are shown in Figure 4. As shown in Figure 4a to c, the as-prepared SSZ-13 materials showed the typical CHA phase [16,17]. To clarify the effect of the synthesis conditions on the crystallinity of SSZ-13, the relative crystallinities for each Si/Al ratio were independently normalized and are displayed in Figure 4d to f. The results showed that the crystallinity of the as-prepared SSZ-13 decreased as the pH values at fixed molar ratios of SDAs/Si or Na_2_O/Si as the TMAda-OH (25 wt%) and NaOH (48 wt%) solution used in our work showed strong alkalinity. Clearly, to determine the best synthesis condition, the results of the chemical composition of crystalline SSZ-13 should be considered, and the results are shown in Table 2. For the synthesis of SSZ-13 with Si/Al = 8, the Si/Al ratios of S-1 and S-2 showed a great difference from those of the prepared gel (eight), which illustrated that the molar ratio of SDAs/Si = 0.15 was not enough to ensure that all the Al ions entered the CHA framework. Taking the relative crystallinity into account, the synthesis condition of SSZ-13 with Si/Al = 8 was the best choice for Sample 3 (S-3). Furthermore, for the synthesis of SSZ-13 with larger Si/Al ratios (15 and 25), it was easy to claim that the synthesis conditions of S-5 and S-12 were the optimal ones when considering similar relative crystallinities (S-5 and S-7 for Si/Al = 15; S-12 and S-15 for Si/Al = 25) and real Si/Al ratios at the same time.

At this point, we obtained the optimal condition for the synthesis of SSZ-13 in wide range of Si/Al ratios. Within this optimal condition, the processes of how the different sizes of SSZ-13 with a uniform size distribution were obtained will be illustrated in the following part.

### 3.2. Synthesis of Different Sizes of SSZ-13 Materials

According to the former studies [7,18,19], the change of the grain size is related to the relative rate of nucleation and crystallization growth, and the supersaturation of the precursor is a critical factor influencing the growth rate. Hence, the supersaturation in the synthesized gel with a simple method should be considered a priority, like directly changing the pH or the water content or the addition of the seed in the synthesized gel. Therefore, each variable was changed independently to better investigate how these variables influenced the grain size of SSZ-13.

**Figure 5:**
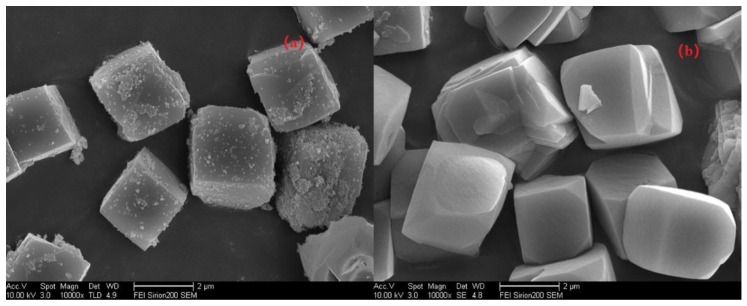
SEM images of S-12 (**a**) and S-14 (**b**).

First, let us quickly review the experiments to regulate the pH values via adding different Na_2_O/Si rations in the gel when synthesizing Si/Al = 25 SSZ-13 as an example. To gain more insight into the morphological changes while altering the pH values, the resultant SSZ-13s of S-12 and S-14 were characterized using SEM, and the results are shown in Figure 5. The higher pH values resulted in high supersaturation of the precursor, which would cause smaller grain sizes; whereas, two samples showed cubic crystals with a similar average size (~2 μm), which illustrated that the grain size was not sensitive to the pH value. This is easy to understand because the range of suitable pH values for SSZ-13 synthesis is narrow (13.0~13.8). Thus, it is reasonable believe that altering the pH value was not a feasible approach to obtain SSZ-13s with different sizes.

**Figure 6:**
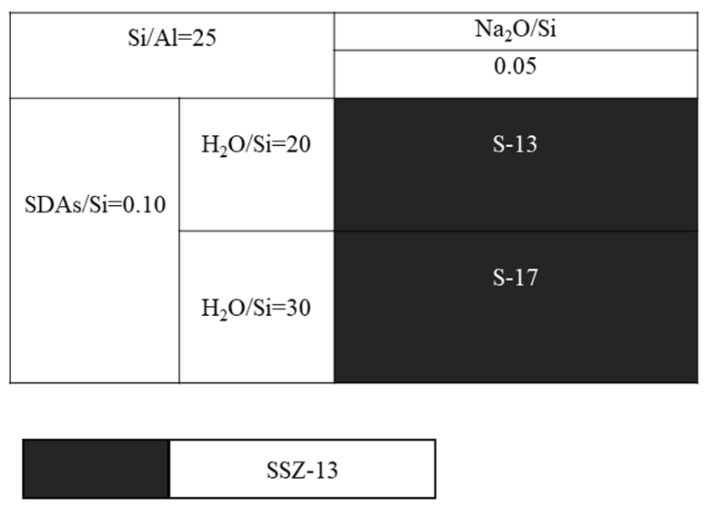
SEM images of S-13 and S-17.

**Figure 7:**
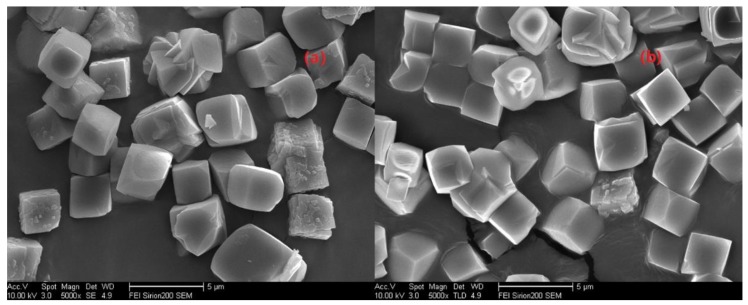
The crystallization of SSZ-13 with Si/Al = 25 as a function of the H_2_O/Si ratios in alkaline media. (**a**) S-13; (**b**) S-17.

Next, another question is whether the molar ratio of H_2_O/Si could alter the grain size of the as-prepared SSZ-13, and this will be answered by running a series of comparative experiments that only change the H_2_O/Si molar ratios. In this case, SSZ-13 with Si/Al = 25 was still chosen. Additionally, to avoid large fluctuations of the pH values caused by more water addition, resulting in no crystallization [20], we preferred to use more NaOH, and the detailed experiments are shown in Figure 6. Again, changing the water content could adjust the supersaturation of the precursor, and this would in turn make the grain size of the as-prepared SSZ-13 change. However, the result of SEM in Figure 7 shows that the increase in H_2_O/Si ratios could make the SZZ-13 material crystallize, and the morphology of these two samples did not show much difference, especially the grain size. Notably, if these samples were further examined by XRF (Table 3), the Si/Al ratio of S-17 was 50.6, which was twice as big as the feed ratio (15). Considering all these facts, changing the water content is not also a universal method to adjust the grain size of SSZ-13. At this point, adjusting the pH value and water content was not a practical solution to alter the grain size.

In the end, the method of adding the seed into the precursor gel was applied to illustrate how this approach gradually achieved the final goal of this work. It is well known that seed addition, usually used for the as-prepared zeolite, can act as a crystal nucleation site to synthesize zeolites [21,22]. In the same way, the as-prepared SSZ-13 could be used as the seed, and a greater amount of seed addition should result in the equal increase of the crystal nucleation sites in the gel. As the reactant is limited in the synthesized gel, introducing more crystal nucleation sites amounts to helping to generate a smaller crystal size [7]. 

**Figure 8:**
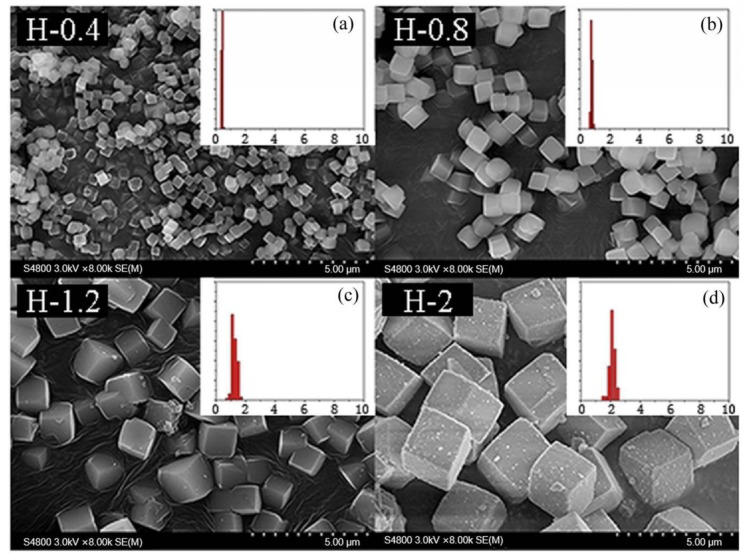
The SEM results of SSZ-13 with different sizes (Si/Al = 25). (**a**) H-0.4; (**b**) H-0.8; (**c**) H-1.2; (**d**) H-2. Synthesized gel compositions: 1 SiO_2_: 0.02 Al_2_O_3_: 0.025 Na_2_O: 0.1 SDA: 20 H_2_O.

Drawing on this idea, the grain size of the as-prepared SSZ-13 materials was determined by using the above optimized Si/Al = 25 SSZ-13s (S-12) as the seeds. Now, two points need special attention. One is that seeds having the same Si/Al ratio as the final sample should be used because this would effectively prevent the samples having different Si/Al ratios at the inner and outer layers [12]. The other is that SSZ-13 should be ground before being added into the precursor gel. In fact, different amounts of SSZ-13 without milling as seeds were used to change the size of SSZ-13 materials with Si/Al = 25 in our pre-experiments. However, the grain size of the as-prepared SSZ-13 with Si/Al = 25 could only be reduced from 2 to 1.5 μm when the amount of seeds added reached 40 wt%, which showed that a moderating effect of size was not obvious if SSZ-13 without milling was used. A reasonable explanation is that SSZ-13s without milling were too large compared with the crystal nucleus, and hence, these were not suitable sites to allow the reactants to continue to crystallize. If the optimized SSZ-13s (S-12) were ground in a ball mill for 0.5 h before synthesis, the size of the as-prepared SSZ-13s could be effectively controlled from 2 to 0.4 μm (Figure 8) when the additive amounts increased from 0% to 1% (Table 4). It is also important to highlight that the size distribution of every obtained sample with cubic-like particles was uniform. Moreover, compared to SSZ-13 synthesis with unground seed, the amount of ground seed addition could be effectively decreased (from 40% to 1%). The obtained results suggested that a suitable approach for the adjustment of the size was using the ground SSZ-13 materials as seeds before crystallization.

**Figure 9:**
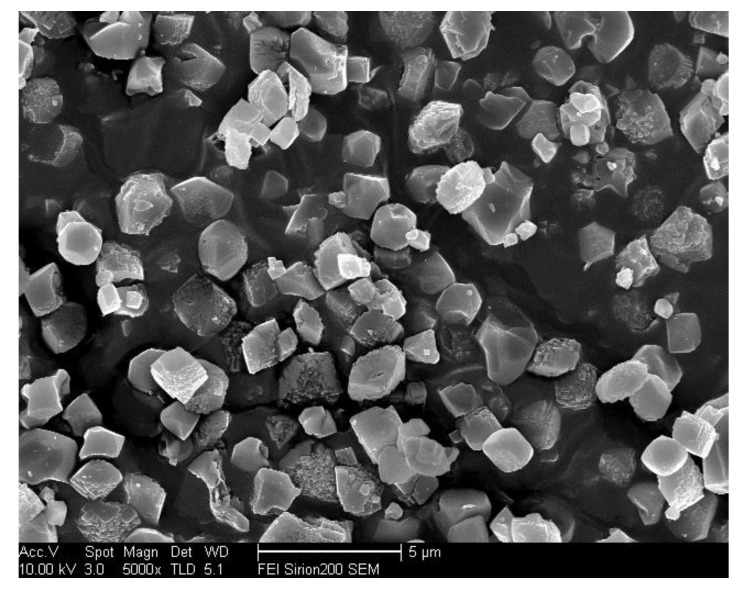
The SEM result of the as-prepared SSZ-13 using ground seeds with 1 h grinding.

**Figure 10:**
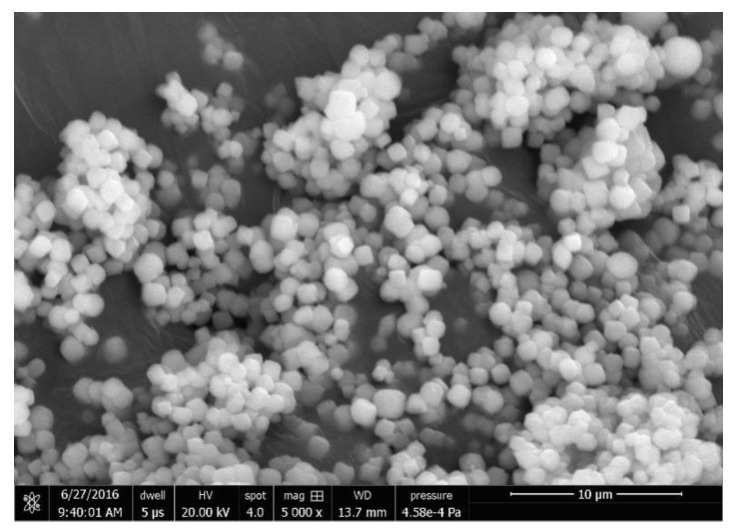
The SEM results of 0.5 h of seed grinding.

Could extending the grinding time improve the morphology of the samples? This question could be answered by running a series of comparative experiments using 1% ground seed with 1h of grinding under identical synthesis conditions as that of the sample ground for 0.5 h. The SEM results in Figure 9 show that a longer grinding time led to inhomogeneous crystals of SSZ-13 materials, manifesting that a longer grinding time was not helpful to obtain a good morphology of the as-prepared SSZ-13. As shown in Figure 10, after 0.5 h of grinding, the uniformity of the size distribution of the as-prepared SSZ-13 materials should be relative to the degree of uniformity of the ground seeds. Here, the optimum condition for obtaining the variable grain sizes of SSZ-13 (Si/Al = 25) with a uniform size distribution was finally achieved. 

**Figure 11:**
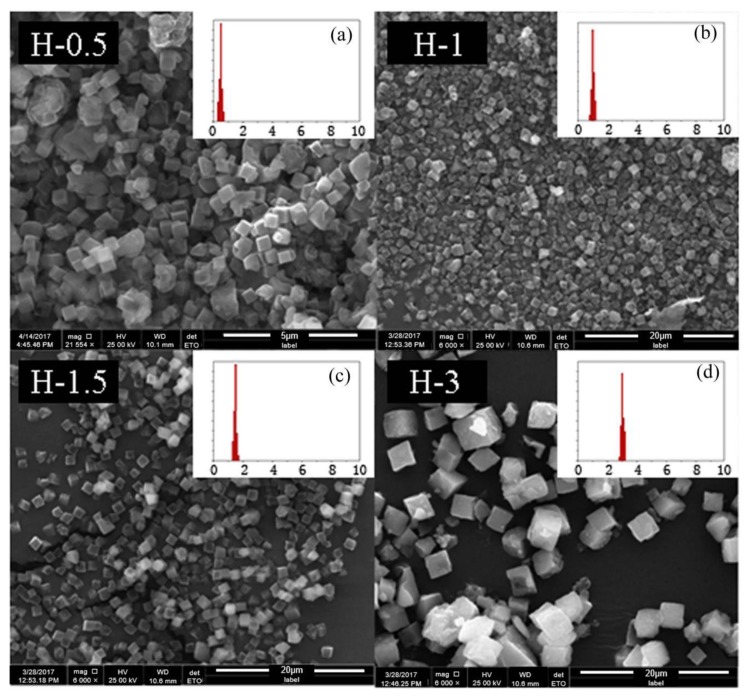
The SEM results of SSZ-13 with different sizes (Si/Al = 8). (**a**) H-0.5; (**b**) H-1; (**c**) H-1.5; (**d**) H-3. Synthesized gel compositions: 1 SiO_2_: 0.06 Al_2_O_3_: 0.05 Na_2_O: 0.2 SDA: 20 H_2_O.

**Figure 12:**
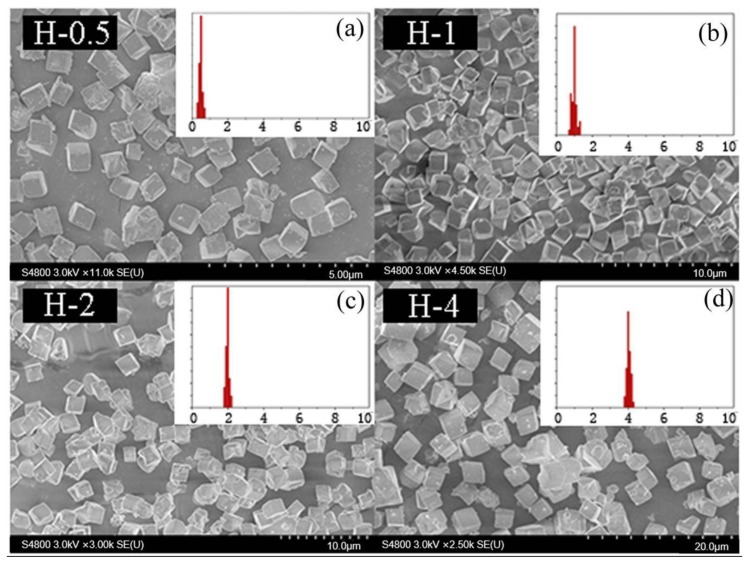
The SEM results of SSZ-13 with different sizes (Si/Al = 15). (**a**) H-5; (**b**) H-1; (**c**) H-2; (**d**) H-4. Synthesized gel compositions: 1 SiO_2_: 0.03 Al_2_O_3_: 0.05 Na_2_O: 0.1 SDA: 20 H_2_O.

To further confirm that the above optimal condition was a universal method, SSZ-13s with different Si/Al ratios were also synthesized using ground seed under the optimized synthesis formula in Section 2.1. As shown in Figure 11 and Figure 12, the different sizes and uniform size distribution of the as-prepared SSZ-13s with Si/Al ratios equal to eight and 15 could also be obtained, verifying that using ground seed was a practical solution to deal with the grain size of SSZ-13 materials.

**Figure 13:**
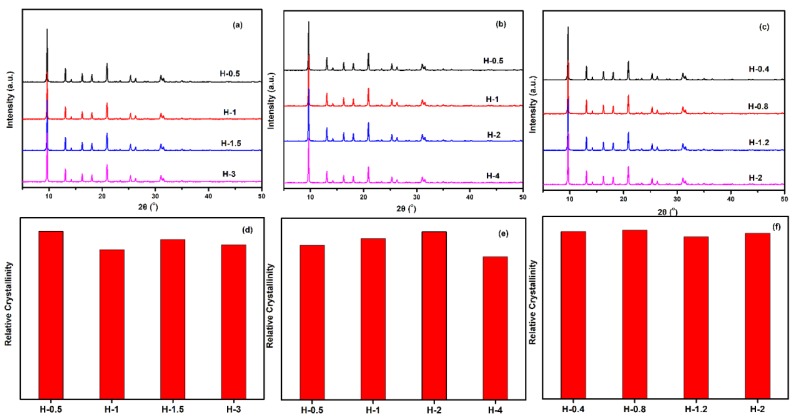
XRD pattern (top) and relative crystallinity (bottom) of SSZ-13s with different sizes. (**a**,**d**) for Si/Al = 8 SSZ-13 materials; (**b**,**e**) for Si/Al = 15 SSZ-13 materials; (**c**,**f**) for Si/Al = 25 SSZ-13 materials. Notes: The relative crystallinities for each Si/Al ratio were independently normalized.

**Figure 14:**
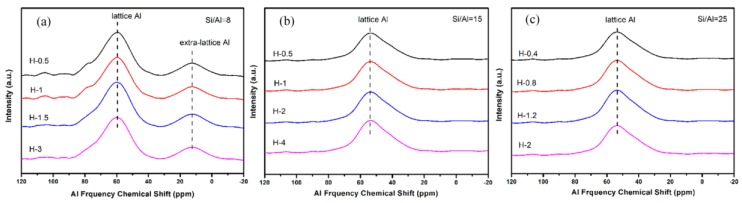
^27^Al NMR results of SSZ-13 with different sizes at different Si/Al ratios. (**a**) Si/Al = 8; (**b**) Si/Al = 15; (**c**) Si/Al = 25.

To gain a better understanding of the crystallinity and chemical compositions of the above samples with different sizes, the samples were further measured by XRD and XRF. From the results of Figure 13 and Table 5, all synthesized samples had a similar relative crystallinity and chemical composition under each Si/Al ratio. The next question for the obtained material was whether the as-prepared SSZ-13s with different grain sizes under the special Si/Al ratio also had a similar acidity. To confirm the above suggestion, ^27^Al NMR measurements of all samples were performed because the acidity of SSZ-13s resulted from Al atom insertion into the CHA structure [1]. As shown in Figure 14, the samples with different grain sizes had basically the same intensity for the Al peaks (lattice Al and extra-lattice Al), which manifested that the micro-chemical environments of Al were identical. Considering that the identical chemical environment of Al resulted in the same acidity [6], the above results indicated that the different sizes of SSZ-13s with a similar Si/Al ratio had similar acidity. Moreover, the above results also indicated that adding different amounts of seeds alone adjusted the size of the as-prepared SSZ-13 materials, rather than other features. Again, adding the different amounts of ground seeds was a helpful approach to gain different sizes of SSZ-13 crystals. 

Here, as adding ground seeds is an easy method to carry out in industry, this approach is very suitable for industrial production of SSZ-13 crystals. Additionally, in combination with the successful case of synthesizing different sizes of SAPO-34 with an identical method, it is reasonable to believe that adding the ground seeds may also prove a useful method to synthesize other types of zeolites.

## 4. Conclusions

The synthesis of SSZ-13 materials with variable grain sizes and a uniform size distribution was systematically investigated, and the detailed synthesis process was given. The main conclusions of this work are listed in the following:

1. The SDA content was an essential factor to determine the Al entering the CHA framework, and hence, the molar ratios of SDAs/Si could not be lower than 0.1 in our work.
2. The molar ratios of Na_2_O/Si also affected the SSZ-13 crystallinity as too high or too low contents did not benefit the synthesis of SSZ-13. The pH value centered between 13 and 13.8 was an appropriate condition for crystallization.
3. Due to the limitation of the synthetic pH value, the change of supersaturation via turning alkaline and water content was not suitable to generate different grain sizes of SSZ-13s.
4. Under fixed compositions of the precursor gel, the addition of different contents (0 to 1 %) of ground SSZ-13 as seeds was an effective and easy way to synthesize different grain sizes (0.4 to 4 μm) of SSZ-13 materials with a uniform size distribution with different Si/Al ratios (8, 15, and 25).
5. Our study suggested that adding seeds was very suitable for the industrial production of SSZ-13 crystals and may also be a useful method to synthesize other types of zeolites in the future.

## Figures and Tables

**Table 1:** The designed experimental variables ^1^.

Variables	Values
TMAda-OH/Si ^2^	0.05, 0.1, 0.15, 0.2
Na_2_O/Si	0.025, 0.05, 0.1, 0.15

^1^ Si/Al = 8, 15, 25; T = 160 °C, t = 96 h, H_2_O/Si = 20; TMAda-OH = *N,N,N*-trimethyl-1-adamantammonium hydroxide; Na_2_O = 2NaOH; ^2^ 0.1, 0.15, and 0.2 for Si/Al = 8 and 15 SSZ-13s’ synthesis; 0.05, 0.1, and 0.15 for SSZ-13s’ synthesis with Si/Al = 25.

**Table 2:** Si/Al ratios for the obtained SSZ-13s.

Sample	Si/Al	Sample	Si/Al
S-1	11.8	S-9	15.1
S-2	11.3	S-10	14.6
S-3	8.2	S-11	15.2
S-4	8.9	S-12	24.7
S-5	15.3	S-13	25.6
S-6	15.2	S-14	23.8
S-7	15.6	S-15	24.5
S-8	14.9	S-16	25.2

**Table 3:** Si/Al ratios for the obtained SSZ-13s.

Sample	Si/Al
S-13	25.6
S-17	50.6

**Table 4:** Relationship between seed addition and the obtained particle size.

The Size of As-Prepared SSZ-13 (μm)	The Amount of Seed Addition (%)
2	0
1.2	0.01
0.8	0.1
0.4	1

**Table 5:** Si/Al ratios for the obtained SSZ-13s with Si/Al = 8, 15, and 25.

Samples with Low Si/Al	Si/Al	Samples with Middle Si/Al	Si/Al	Samples with High Si/Al	Si/Al
H-0.5	7.8	H-0.5	14.5	H-0.4	23.6
H-1	8.3	H-1	15.1	H-0.8	24.8
H-1.5	7.6	H-2	14.2	H-1.2	23.9
H-3	8.5	H-4	13.4	H-2	24.5
